# Quantitative proteomic and functional comparison of extracellular vesicles from multiple adipose tissue mesenchymal stem cell donors

**DOI:** 10.20517/evcna.2025.173

**Published:** 2026-03-30

**Authors:** Kyong-Su Park, Dae Hyun Ha, Jun Ho Lee, Negar Ordouzadeh, Markus Bergqvist, Hyun Ju Lee, Ella Shin, Byong Seung Cho, Jan Lötvall

**Affiliations:** ^1^Krefting Research Centre, Institute of Medicine, Sahlgrenska Academy, University of Gothenburg, Gothenburg 40530, Sweden.; ^2^ExoCoBio Exosome Institute (EEI), ExoCoBio Inc., Seoul 08594, Republic of Korea.; ^3^Department of Life Science and Research Institute of Natural Sciences, College of Natural Sciences, Hanyang University, Seoul 04763, Republic of Korea.; ^4^Exocure Sweden AB, Gothenburg 41126, Sweden.; ^#^These authors contributed equally to this work.

**Keywords:** Extracellular vesicles, exosomes, mesenchymal stem cells, membrane proteins, consistency, anti-inflammation, therapy

## Abstract

**Aim:** Extracellular vesicles (EVs) released by mesenchymal stem cells (MSCs), known as MSC-EVs, have gained attention as potential treatments owing to their immunomodulatory functions. Despite growing clinical interest, donor-to-donor inconsistencies remain key challenges for standardizing MSC-EV production under good manufacturing practice (GMP) conditions. This work aimed to systematically compare the molecular and functional consistency of EVs derived from three independent human adipose tissue-MSC donors.

**Methods:** GMP-grade EVs were initially isolated using tangential flow filtration on a large scale and then characterized by multiple biophysical analyses. To characterize the protein composition of EVs across batches, quantitative proteomic analysis was performed using tandem mass tags and mass spectrometry. For functional validation, an *in vitro* macrophage inflammation assay was conducted by treating natural lipopolysaccharide-stimulated cells with EVs, and cytokine levels were measured using enzyme-linked immunosorbent assays (ELISA).

**Results:** Quantitative proteomic profiling identified 2,615 proteins, of which 84%-94% were not significantly changed across batches, highlighting a robust core proteome. Notably, 361 membrane-associated proteins were consistently conserved, including transporters, adhesion molecules, and signaling receptors, implicating these components in EV-mediated intercellular communication and immunomodulation. Functional analysis using an *in vitro* macrophage inflammation model demonstrated that all EV batches reproducibly suppressed pro-inflammatory cytokine production in a dose-dependent manner, with no significant inter-batch differences.

**Conclusion:** Collectively, these findings indicate that MSC-EVs maintain both molecular and functional stability across different donors, and that a conserved proteomic signature underlies their reproducible anti-inflammatory activity. This study provides a foundation for establishing standardized quality criteria and advancing MSC-EVs toward clinical therapeutic applications.

## INTRODUCTION

Mesenchymal stem cells (MSCs) have emerged as a promising source of therapeutic secretome, which mimics the potent anti-inflammatory and regenerative properties of the parent cells^[1,2]^. Recent evidence suggests that many biological effects of MSCs are mediated not by direct cell engraftment, but by the secretion of extracellular vesicles (EVs)^[3]^. These nano-sized vesicles, which contain diverse bioactive molecules including proteins, lipids, and nucleic acids, play a critical role in intercellular communication and the regulation of immune responses^[4,5]^. Increasing preclinical and clinical data have demonstrated the therapeutic potential of MSC-derived EVs, particularly their capacity to modulate inflammation and tissue injury^[6]^.

To advance the clinical application of EVs released by MSCs (MSC-EVs), it is essential to establish robust and scalable production systems that comply with good manufacturing practice (GMP) standards^[7]^. GMP-compliant manufacturing ensures product safety, efficacy, and reproducibility, which are crucial for therapeutic consistency and regulatory approval. Nevertheless, variations in the MSC tissue source can affect the molecular composition and bioactivity of the produced EVs, presenting a major obstacle to the standardization of MSC-EV products^[8,9]^. Therefore, understanding and validating the consistency of EV characteristics across different production batches and donor sources are key steps toward ensuring their clinical reliability.

In this study, we aimed to assess donor-to-donor consistency in MSC-derived EVs by comparing vesicles obtained from three independent MSC donors under GMP conditions. Specifically, we focused on the characterization of membrane-associated proteins, which are known to play pivotal roles in EV-mediated biological functions such as cell signaling and immune modulation^[10]^. This focus was motivated by our prior work showing that MSC-derived EVs retained therapeutic activity after osmotic depletion of cytosolic contents^[11]^, indicating that surface-associated components are likely critical mediators of EV function. Furthermore, we evaluated the therapeutic potential of different EV batches using an *in vitro* macrophage inflammation model to determine whether consistent protein profiles correlate with anti-inflammatory efficacy. These analyses provide insights into the shared molecular features of MSC-EVs across donors and support the development of standardized quality criteria for their clinical use.

## METHODS

### Cell cultures

Subcutaneous adipose tissue samples from three healthy donors were provided by ExoCoBio Inc. (Seoul, Republic of Korea). Sample collection was approved by the Institutional Review Board of CHA University Medical Center (Gyeonggi-do, Republic of Korea; IRB no. CHAMC 2019-05-040-018) and evaluated according to the guidelines of the Korean Ministry of Food and Drug Safety (MFDS, Republic of Korea). The three donors were healthy female individuals with a mean age of 26.3 ± 3.7 years and a mean body mass index (BMI) of 25 ± 2.4 [Supplementary Table 1]^[12]^. All these donors were covered by the same ethics approval, originated from the same institution, underwent the same GMP manufacturing workflow, and provided informed consent.

Human MSCs (passage 4) were isolated from adipose tissue under GMP conditions at ExoCoBio Inc., as previously described^[13]^. The cells were cultured in α-minimum essential medium (Invitrogen, Carlsbad, CA) supplemented with 10% fetal bovine serum (FBS), 10 ng/mL basic fibroblast growth factor (bFGF), and 1% Gentamicin. Long-term stability of MSCs from the three donors was confirmed, as no morphological differences or changes in population doublings were observed up to passage nine. MSC donor screening included testing for hepatitis B virus (HBV), hepatitis C virus (HCV), and human immunodeficiency virus (HIV), and sterility testing (endotoxin, mycoplasma, and microbial contamination), all of which met release criteria. Moreover, according to the International Society of Cellular Therapy (ISCT) guidelines, MSC identity was confirmed by flow cytometry showing high expression of CD29, CD90, and CD105 and very low expression of CD31, CD45, and human leukocyte antigen - DR isotype (HLA-DR) in all three cell batches. In addition, tri-lineage differentiation assays demonstrated adipogenic, osteogenic, and chondrogenic potential, confirming comparable baseline potency among different MSC batches [Supplementary Table 1].

RAW264.7 cells were cultured in Dulbecco’s modified Eagle’s medium (HyClone, Logan, UT) supplemented with 10% FBS, 100 U/mL penicillin, and 100 µg/mL streptomycin. All cell lines were maintained at 37 °C in a humidified incubator with 5% CO_2_.

### Isolation of MSC-EVs

MSCs maintained under continuous GMP conditions in liquid nitrogen freezers were thawed from a single stock and cultured through passage 8. At passage 8, MSCs were seeded at a density of 6 × 10^3^ cells/cm^2^ and maintained in α-MEM supplemented with 10% FBS, 10 ng/mL bFGF, and 1% gentamicin for 3 days until reaching approximately 90% confluence. To prepare the conditioned medium (CM) for EV isolation, cells were washed with Dulbecco’s phosphate-buffered saline (DPBS; Invitrogen, Carlsbad, CA) and subsequently cultured in serum-free and phenol red-free α-minimum essential medium (α-MEM) for 24 h. The collected CM was first clarified by filtration through a 0.45-μm polyethersulfone (PES) membrane filter (Sartorius, Göttingen, Germany) to remove cells, apoptotic bodies, and cellular debris. The CM was then concentrated by tangential flow filtration (TFF) using a 500-kDa cutoff membrane (Repligen, Chicago, IL) and exchanged into excipient phosphate-buffered saline (PBS) solution by diafiltration. The resulting MSC-EVs were sterile-filtered through a 0.22-μm PES membrane filter (Sartorius, Göttingen, Germany) and stored at -80 °C until further use.

### Transmission electron microscopy

EVs were visualized using transmission electron microscopy (TEM) following negative staining. Briefly, glow-discharged, formvar- and carbon-coated 200-mesh copper grids (Electron Microscopy Sciences, Hatfield, PA) were incubated with EV samples for 5 min. After gentle rinsing in distilled water, the grids were fixed with 2.5% glutaraldehyde in PBS and subsequently negatively stained with 2% uranyl acetate for 1.5 min. Imaging was carried out on a LEO 912AB Omega TEM (Carl Zeiss SMT, Oberkochen, Germany) at 120 kV using a Veleta charge-coupled device (CCD) camera (Olympus-SiS, Stuttgart, Germany).

### Nanoparticle tracking analysis

EVs were diluted in PBS, and particle concentration and size distribution were measured using a ZetaView particle analyzer (Particle Metrix GmbH, Meerbusch, Germany). Each sample was analyzed in triplicate, with data collected from two stationary positions and five measurements per position. For all recordings, the camera sensitivity was adjusted to 70. ZetaView software (version 8.2.30.1) was used for data acquisition and subsequent analysis.

### Nano-flow cytometry

The surface protein expression of EVs was analyzed using a Flow NanoAnalyzer (NanoFCM Inc., Nottingham, UK) following the manufacturer’s protocol. Diluted EV samples were exposed to pre-diluted fluorescent antibodies and left to incubate for 40 min at 25 °C, shielded from light. The antibodies used were fluorescein isothiocyanate (FITC)-conjugated anti-human CD9 (BD Biosciences, Franklin Lakes, NJ), FITC-conjugated anti-human CD63 (BD Biosciences), and FITC-conjugated anti-human CD81 (BD Biosciences). Following incubation, the samples were further diluted in DPBS and analyzed using the Flow NanoAnalyzer. Instrument parameters were set as follows: laser power of 10 mW at 488 nm and 20 mW at 638 nm, sampling pressure of 1.0 kPa, recording time of 1 min, side scatter (SS) decay of 10%, and a 525/40 filter for FITC detection. Data acquisition and analysis were performed using NanoFCM Professional Software (version 2.0) to determine the percentage of positive particles and particle concentration.

### Liquid chromatography-tandem mass spectrometry analysis

Aliquots with 30 µg of protein from three independent batches of each vesicle sample underwent trypsin digestion according to the filter-aided sample preparation (FASP) procedure^[14]^. The samples were reduced [10 mM dithiothreitol (DTT)] and alkylated (20 mM iodoacetamide). Proteins were precipitated on the Sera-Mag™ SpeedBeads (Cytiva) by acetonitrile, washed and dried at room temperature. Beads were resuspended in 100 mM 4-(2-hydroxyethyl)-1-piperazineethanesulfonic acid (HEPES) and proteins were digested with Trypsin/Lysyl endopeptidase (Lys-C) mix [1:25] overnight and additional trypsin [1:50] for an extra 2 h. Beads were removed, and peptides were labelled using tandem mass tag (TMT) pro 18-plex isobaric mass tagging reagents (Thermo Fisher Scientific) according to the manufacturer’s instructions. The labeled samples were pooled and purified by high protein and peptide recovery (HiPPR) Detergent Removal Resin and Pierce™ and Peptide Desalting Spin Columns (both from Thermo Fisher Scientific). The TMT-set was fractionated using basic reversed-phase chromatography (bRP-LC, pH10) over 70 min into 24 fractions. Each fraction was analyzed on Orbitrap Eclipse™ Tribrid™ mass spectrometer equipped with the high-field asymmetric waveform ion mobility spectrometry (FAIMS) Pro ion mobility system interfaced with nano liquid chromatography (nLC) 1200 liquid chromatography system (all Thermo Fisher Scientific). Peptides were separated on a C18 40 cm column over 85 min and data were acquired with synchronous precursor selection multi-stage mass spectrometry 3 (SPS MS3) method. The mass spectrometry (MS) proteomics data has been deposited to the ProteomeXchange Consortium (http://proteomecentral.proteomexchange.org) via the PRIDE partner repository with the data set identifier PXD071815.

### Database search

Protein identification and quantification were performed using Proteome Discoverer software (version 2.2; Thermo Fisher Scientific, Waltham, MA) against the Homo sapiens SwissProt database. Mascot (v2.5.1; Matrix Science, London, UK) served as the search engine with precursor and fragment ion tolerances of 10 ppm and 0.6 Da, respectively. A single missed tryptic cleavage was tolerated. Methionine oxidation was specified as a variable modification, and cysteine methylthiolation as fixed. Peptide-spectrum match (PSM) validation was conducted using the Percolator algorithm, with a stringent false discovery rate (FDR) cutoff of 1%. Protein classification and pathway enrichment analyses were performed using the PANTHER Classification System (http://pantherdb.org/), and Gene Ontology (GO) enrichment analysis was conducted with the DAVID Bioinformatics Resources (https://david.ncifcrf.gov/). Additionally, membrane proteins were not experimentally validated but were identified using curated subcellular-localization databases from UniProt.

### Sodium dodecyl sulfate–polyacrylamide gel electrophoresis

EV proteins were separated on a TGX Stain-Free FastCast acrylamide gel (Bio-Rad Laboratories, Hercules, CA) at 180 V for 30 min, followed by visualization of total proteins with the ChemiDoc MP imaging system (Bio-Rad Laboratories, Hercules, CA).

### Isolation of outer membrane vesicles derived from *Escherichia coli*

Bacterial cultures were centrifuged at 6,000 × *g* for 20 min at 4 °C to remove cells and large debris. After filtering the supernatant through a 0.45-μm vacuum filter, the supernatant was concentrated using a Vivaflow 200 ultrafiltration device (100-kDa cutoff; Sartorius, Göttingen, Germany). The concentrated sample underwent ultracentrifugation at 150,000 × *g* for 3 h at 4 °C, and the pellet containing bacterial vesicles was resuspended in PBS for further experiments.

### RAW264.7 cytokines *in vitro*

Cells in 24-well plates were stimulated with 100 ng/mL outer membrane vesicles (OMVs) for 3 h to trigger pro-inflammatory cytokine expression. Subsequently, cells were treated with varying concentrations of EVs (3 × 10^8^, 1 × 10^9^, or 3 × 10^9^ particles/mL). After 16 h of incubation, the culture supernatants were collected, and the levels of pro-inflammatory cytokines were determined with DuoSet ELISA (enzyme-linked immunosorbent assay) Kits (R&D Systems, Minneapolis, MN) according to the provided protocol.

### Statistical analysis

Data are presented as mean ± standard error of the mean (SEM). Comparisons among multiple groups were performed using one-way analysis of variance (ANOVA) with Tukey’s post hoc test. For comparisons involving two independent variables, two-way ANOVA was performed, followed by Tukey’s multiple comparison test. Statistical analyses were conducted using GraphPad Prism 10 (GraphPad Software Inc., San Diego, CA). *P* < 0.05 was considered to be significant.

## RESULTS

### MSC-derived EVs exhibit conserved biophysical and molecular features across different batches

**Figure 1:**
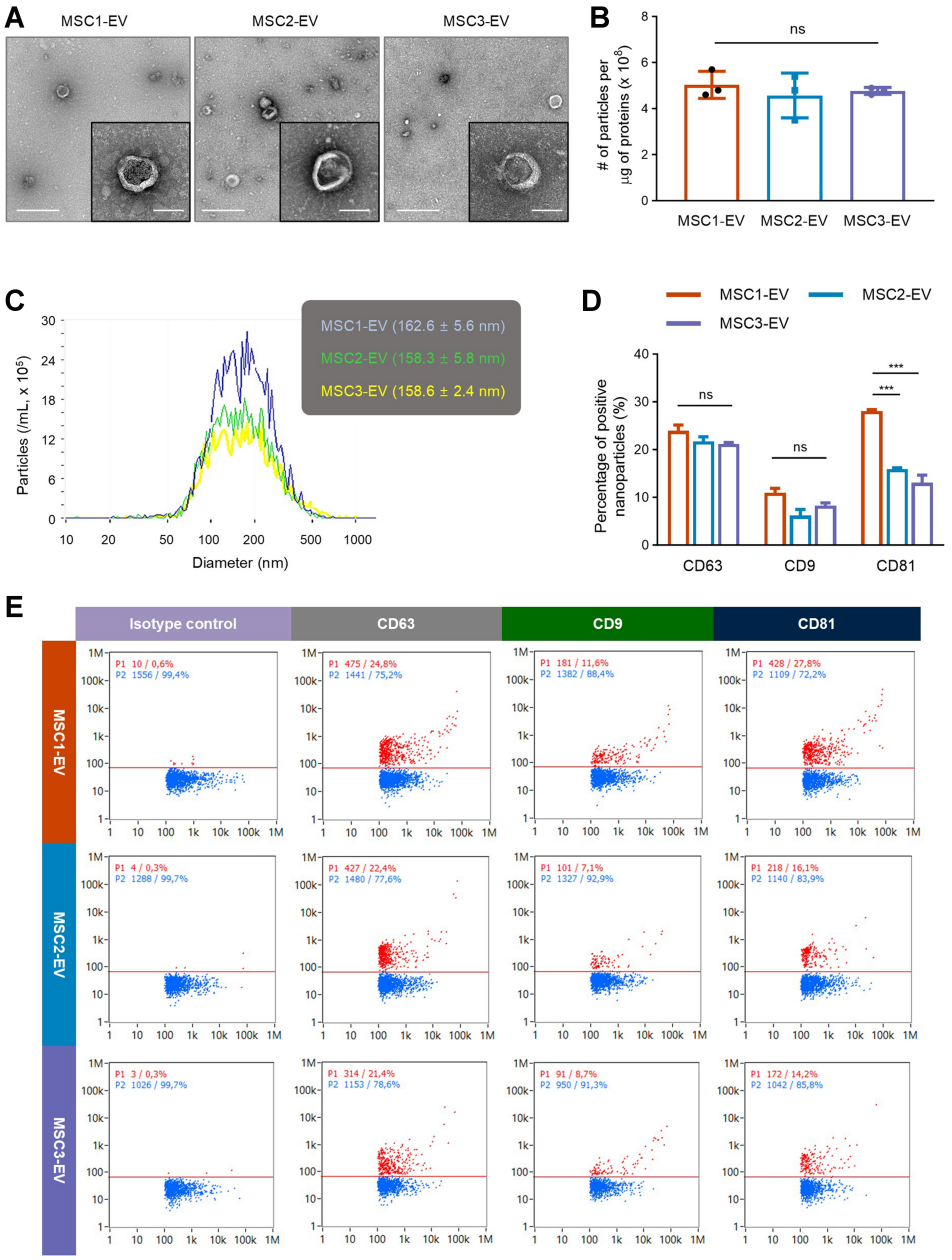
Morphology and marker expression of EVs from different MSC donors. (A) Representative electron microscopy images showing the characteristic vesicular structure across EV batches. The left and right scale bars represent 500 and 100 nm, respectively, in each sample image; (B) The number of particles per one microgram of vesicular proteins (*n* = 3); (C) The size distribution of different EV batches measured by nanoparticle tracking analysis; (D) Nano-flow cytometry analysis showing the percentage of vesicles positive for CD63, CD9, or CD81 across different EV batches (*n* = 2); (E) Representative nano-flow cytometry analysis of three EV batches. Scatter plots show particle populations, with red indicating vesicles positive for a specific tetraspanin protein and blue indicating negative vesicles. The X- and Y-axes represent side scatter height and fluorescence intensity, respectively. Throughout, the data are presented as the mean ± SEM. ^***^P < 0.001; ns, not significant, by one-way ANOVA with Tukey’s post hoc test (B) or two-way ANOVA with Tukey’s post hoc test (D). EVs: Extracellular vesicles; MSC: mesenchymal stem cell; SEM: standard error of the mean; ANOVA: analysis of variance.

GMP-grade MSCs were successfully isolated from adipose tissues of three healthy donors, and EVs were subsequently purified from their conditioned media using TFF and filtration. As shown in Figure 1A, TEM analysis revealed that EVs from all three MSC donors exhibited characteristic nano-size, circular morphology, with no noticeable structural differences among batches. MSC-EV purity was determined using the particle number-to-protein content ratio, revealing comparable purity across the three EV preparations [Figure 1B]. Nanoparticle tracking analysis further confirmed that the average EV diameter ranged from 150 to 170 nm, with similar size distribution profiles between different batches [Figure 1C]. Moreover, nano-flow cytometry was used to examine surface expression of the EV-enriched tetraspanins CD63, CD9, and CD81. The expression levels of CD63 and CD9 remained consistent across all three EV batches, indicating stable molecular profiles among preparations, whereas CD81 expression exhibited some batch-to-batch variation [Figure 1D and E]. Consequently, high-quality MSC-derived EVs were reproducibly isolated across multiple batches for subsequent proteomic and functional analyses.

### Comparative proteomic profiling reveals high consistency of protein composition across different donors

**Figure 2:**
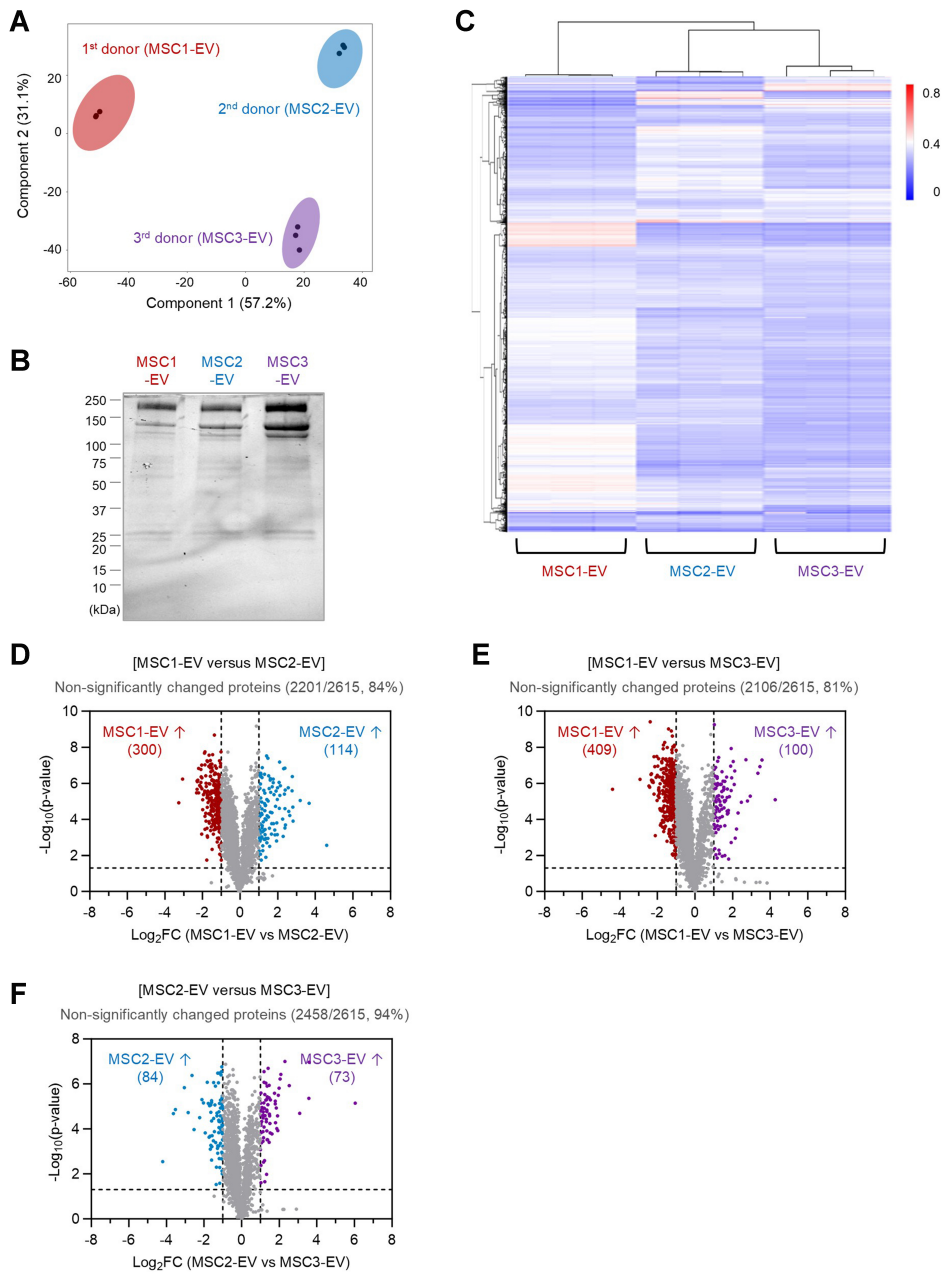
Comparative proteomic analysis of EVs from different MSC donors. (A) PCA of EV proteomes showing clustering of three MSC donor-derived EVs (MSC1-EV, MSC2-EV, and MSC3-EV), each with three replicates; (B) Stain-free SDS-PAGE gel of total EV proteins from all three MSC donors; (C) Partial hierarchical clustering heatmap of relative protein abundances across different EV batches, with each batch represented by three replicates; (D) Volcano plot comparing EV proteomes from different MSC donors (MSC1-EV *vs.* MSC2-EV), representing 300 proteins significantly enriched in MSC1-EV, 114 proteins in MSC2-EV, and 2,201 proteins unchanged (84% of 2,615 total proteins); (E) Volcano plot comparing EV proteomes from different MSC donors (MSC1-EV *vs.* MSC3-EV), representing 409 proteins significantly enriched in MSC1-EV, 100 proteins in MSC3-EV, and 2,106 proteins unchanged (81% of 2,615 total proteins); (F) Volcano plot comparing EV proteomes from different MSC donors (MSC2-EV *vs.* MSC3-EV), representing 84 proteins significantly enriched in MSC2-EV, 73 proteins in MSC3-EV, and 2,458 proteins unchanged (94% of 2,615 total proteins). EVs: Extracellular vesicles; MSC: mesenchymal stem cell; PCA: principal component analysis; SDS-PAGE: sodium dodecyl sulfate-polyacrylamide gel electrophoresis.

To evaluate the similarity of protein composition among different donors of EVs derived from MSCs, a quantitative comparative proteomic analysis was conducted. Principal component analysis (PCA) demonstrated that the first principal component accounted for 57% of the total variance and distinctly separated the samples according to the MSC donor origin, whereas the second component explained 31% of the variance and primarily distinguished biological replicates [Figure 2A]. Each replicate from the same EV batch clustered closely together, indicating strong intra-batch consistency.

A total of 2,615 proteins were identified across all EV samples, and notably, every protein was consistently detected in all batches. The overall protein expression profiles analyzed by SDS-PAGE revealed similar protein band patterns across different EV batches [Figure 2B]. Hierarchical cluster analysis corroborated the PCA results, showing that EV samples first grouped by donor and subsequently by replicate [Figure 2C]. These findings suggest that while EVs derived from different MSC donors possess somewhat distinct protein profiles, replicates within each batch are highly comparable. Notably, the first batch of EVs appeared more distinct from the second and third batches, which in turn exhibited a greater degree of similarity to each other in terms of relative protein abundance. Differential expression analysis, presented as a volcano plot, further quantified the observed differences. Comparison between the first and second EV batches revealed that 300 and 114 proteins were upregulated more than 2-fold in the first and second batches, respectively [Figure 2D]. However, the majority of proteins (2,201; 84% of total) remained unchanged. Similarly, 81% (2,106) of proteins were unaltered between the first and third EV batches [Figure 2E], and a higher proportion (2,458; 94%) were unchanged between the second and third EV batches [Figure 2F]. Overall, these results indicate a high level of conservation in EV protein composition across batches, particularly between the latter two (MSC2-EV and MSC3-EV), consistent with the clustering patterns observed in multivariate analyses.

### Functional enrichment analysis highlights conserved exosomal and transport-related proteins across EV batches

**Figure 3:**
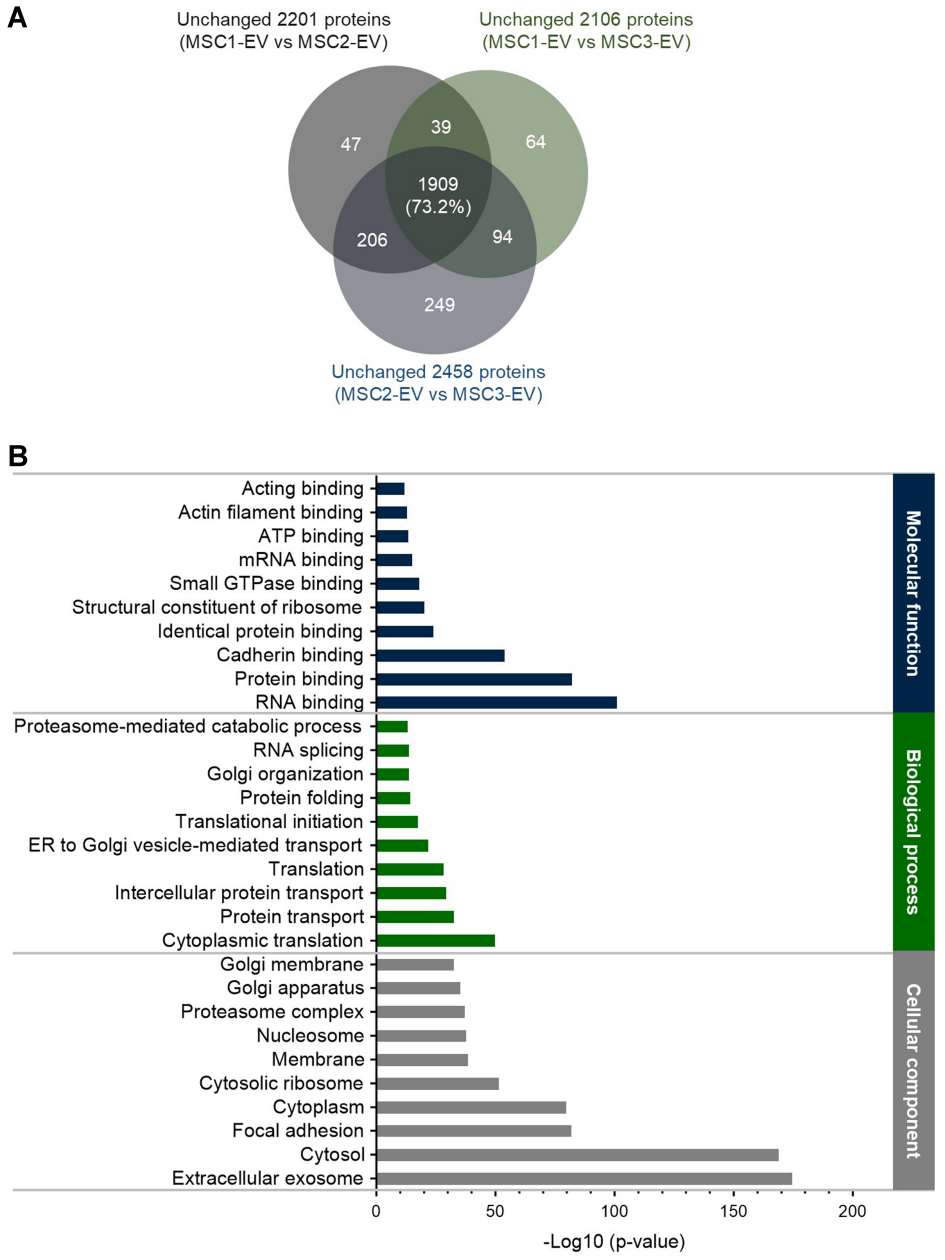
GO analysis of vesicular proteins unchanged across three EV batches. (A) Venn diagram showing the overlap of proteins that remained unchanged (< 2-fold, not significant) in pairwise comparisons of MSC-EVs (MSC1-EV *vs.* MSC2-EV, MSC1-EV *vs.* MSC3-EV, and MSC2-EV *vs.* MSC3-EV). The diagram indicates the proteins consistently stable across all three batches (1,909 proteins, 73.2% of 2,608 total proteins); (B) GO analysis of the common unchanged proteins, categorized by molecular function, biological process, and cellular component. Enrichment analysis highlights proteins associated with exosomes, indicating core EV protein composition conserved across different batches. GO: Gene Ontology; EV: extracellular vesicle; MSC: mesenchymal stem cell; ATP: adenosine triphosphate; mRNA: messenger RNA; ER: endoplasmic reticulum.

Subsequently, GO term enrichment analysis was performed using the sets of unchanged proteins identified between each pair of EV batches (2,201 unchanged proteins for MSC1-EV *vs.* MSC2-EV; 2,106 for MSC1-EV *vs.* MSC3-EV; and 2,458 for MSC2-EV *vs.* MSC3-EV). This analysis aimed to elucidate the conserved functional roles and potential biological origins of the shared proteins among different EV batches. Only proteins that met the quantitative filtering criteria in all relevant comparisons were included, resulting in a combined set of 2,608 proteins, slightly fewer than the total proteome of 2,615 proteins. Venn diagram analysis revealed a substantial overlap of 1,909 (73.2%) proteins common to all three unchanged-protein datasets [Figure 3A], indicating a robust core proteome consistently maintained across MSC-derived EV preparations. Also, when the analyzed common proteins were compared with the top 100 EV proteins listed in the EVpedia database^[15]^, 66 proteins were found to overlap [Supplementary Table 2].

Subcellular localization analysis of these 1,909 common proteins, conducted utilizing the DAVID database, demonstrated that the most significantly enriched annotation was the term “extracellular exosome”, with the lowest *P*-value among all identified categories [Figure 3B]. The result confirms that most conserved proteins correspond to well-established exosomal markers, further validating the purity of the EV preparations. Further, GO analysis of biological processes revealed that the top ten enriched terms were largely associated with transport-related pathways, consistent with findings from other MSC-EV proteomic profiles^[16,17]^. In terms of molecular function, “RNA binding” emerged as the most significantly enriched category, followed by “protein binding” and “cadherin binding”. Notably, cadherin-binding proteins were enriched, reflecting their established roles in cell adhesion and intercellular communication, which are essential for EV-mediated signaling^[18]^. Collectively, these findings suggest that the core, unchanged protein set across MSC-EV batches represents a functionally stable exosomal proteome, primarily involved in molecular transport, RNA regulation, and cell interaction dynamics.

### Membrane proteome analysis identifies conserved functional components across EV batches

**Figure 4:**
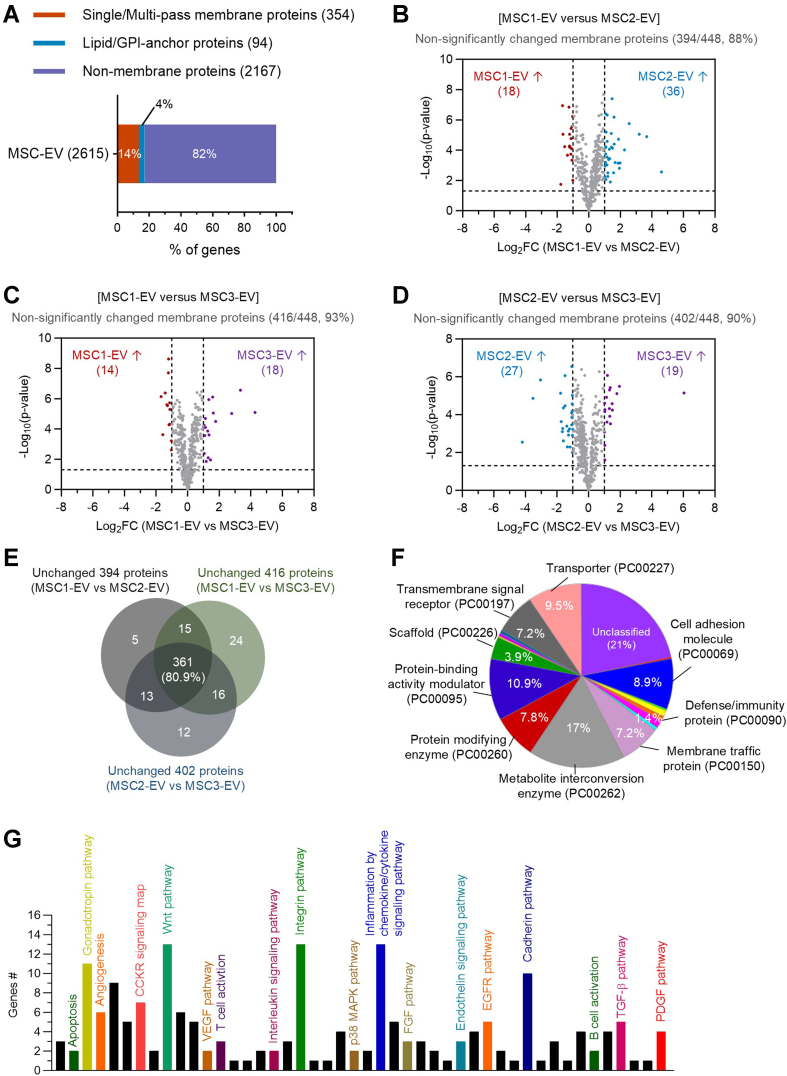
Analysis of conserved EV membrane proteins across donor batches. (A) Composition of membrane proteins in three MSC donor-derived EV proteome, including transmembrane proteins (354, 14%) and lipid/GPI-anchored proteins (94, 4%) of the 2,615 total proteins; (B-D) Volcano plots comparing membrane proteins between different EV batches: MSC1-EV *vs.* MSC2-EV (B; 394 proteins unchanged, 88%), MSC1-EV *vs.* MSC3-EV (C; 416 proteins unchanged, 93%), and MSC2-EV *vs.* MSC3-EV (D; 402 proteins unchanged, 90%); (E) Venn diagram analysis showing overlap of unchanged membrane proteins across the three pairwise comparisons, highlighting 361 proteins consistently stable across different batches; (F) Protein class analysis of the 361 unchanged membrane proteins, annotated by pathway-based functional categories; (G) Panther pathway analysis of the 361 unchanged membrane proteins, identifying signaling pathways potentially associated with EV function. EV: Extracellular vesicle; MSC: mesenchymal stem cell; GPI: glycosylphosphatidylinositol.

Given that membrane proteins play pivotal roles in MSC-EV-mediated therapeutic effects^[11]^, we next performed comparative proteomic analysis focusing specifically on membrane-associated proteins across EV batches. Among the total 2,615 common proteins, 354 were identified as transmembrane proteins and 94 as lipid- or glycosylphosphatidylinositol (GPI)-anchored proteins, representing approximately 18% of the total EV proteome [Figure 4A]. Differential expression analysis using volcano plots revealed that between the first and second EV batches, 18 membrane proteins were upregulated in the first batch and 36 in the second batch by more than 2-fold [Figure 4B]. However, the majority of membrane proteins (394 out of 448; 88%) remained non-significantly altered, a proportion comparable to that observed in the total proteome (84%; Figure 2D). Similarly, 93% of membrane proteins were unchanged between the first and third batches [Figure 4C], and 90% were unchanged between the second and third batches [Figure 4D], indicating high conservation of the membrane protein profile across EV batches.

GO term enrichment analysis was subsequently performed on the unchanged membrane protein sets (394 for MSC1-EV *vs.* MSC2-EV; 416 for MSC1-EV *vs.* MSC3-EV; 402 for MSC2-EV *vs.* MSC3-EV) to explore their functional roles. Venn diagram analysis identified 361 membrane proteins shared across all three comparisons (80.9%, Figure 4E). Functional classification using Panther tools revealed that these conserved membrane proteins were predominantly associated with metabolite interconversion enzymes, transport proteins, receptors for transmembrane signaling, membrane trafficking proteins, and cell adhesion [Figure 4F]. Although a smaller fraction belonged to defense or immunity-related classes, their presence suggests EV-mediated potential immunomodulatory functions. Pathway analysis further indicated that the conserved membrane proteome was involved in integrin signaling, cadherin-mediated adhesion, inflammation, and multiple growth factor-related pathways [Figure 4G], consistent with roles in EV-mediated tissue regeneration, intercellular communication, and anti-inflammatory effects^[19]^. Taken together, these findings demonstrate that MSC-EV membrane proteins are largely conserved across batches, highlighting a stable proteomic signature likely critical for their therapeutic activity.

### MSC-EVs consistently exhibit anti-inflammatory activity against natural LPS-induced inflammation

**Figure 5:**
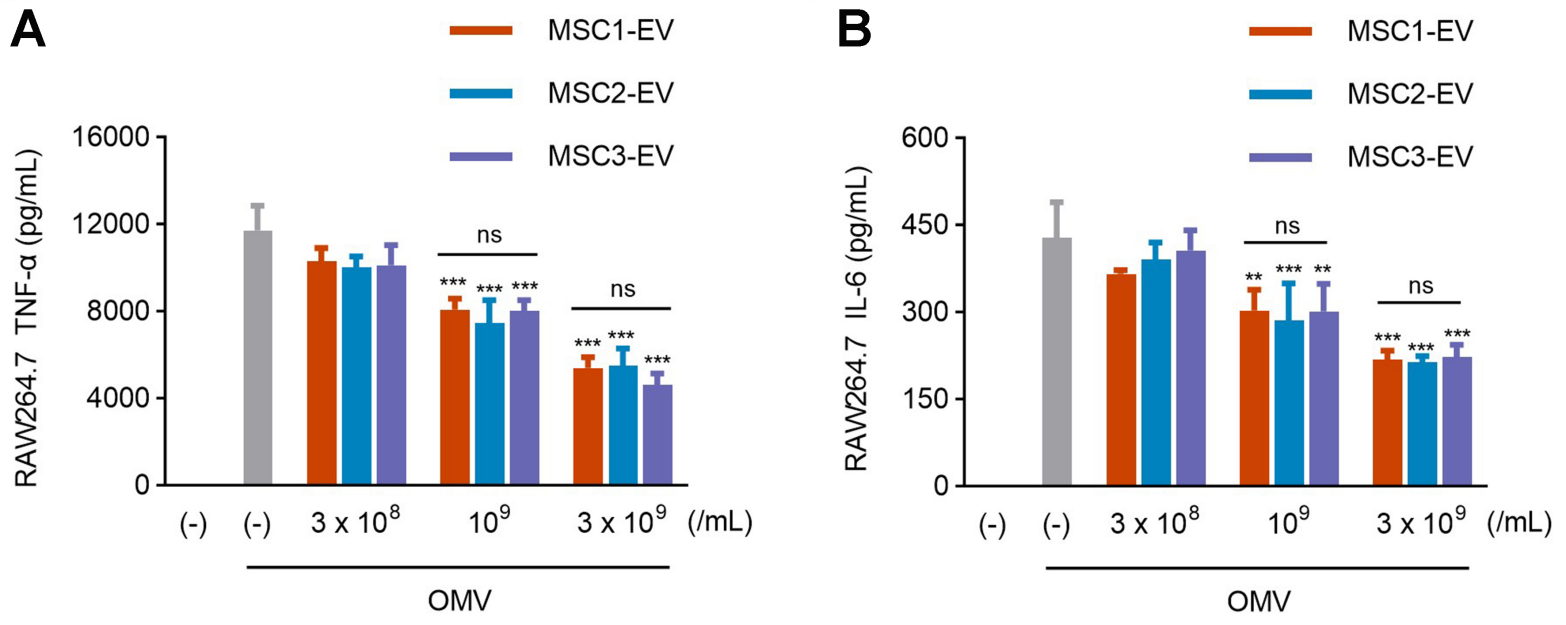
Comparable anti-inflammatory effects of EVs from three MSC donors. (A and B) RAW264.7 cells were treated with OMVs (100 ng/mL) for 3 h, followed by incubation with three different batches of MSC-EVs at various concentrations for 16 h. TNF-α (A) and IL-6 (B) levels were quantified in the conditioned media (*n* = 3). The data are presented as the mean ± SEM. ^**^P < 0.01, ^***^P < 0.001; ns, not significant, by two-way ANOVA with Tukey’s post hoc test. EVs: Extracellular vesicles; MSC: mesenchymal stem cell; OMVs: outer membrane vesicles; TNF-α: tumor necrosis factor-α; IL-6: interleukin-6; SEM: standard error of the mean; ANOVA: analysis of variance.

To assess whether the conserved protein composition observed across MSC-EV batches translates into consistent functional effects, we evaluated batch-to-batch bioactivity using an *in vitro* inflammation model. RAW264.7 macrophage cells were activated with bacteria-derived OMVs, a physiologically relevant lipopolysaccharide (LPS) and a potent stimulator of inflammatory responses^[20]^, and subsequently treated with three independent batches of MSC-EVs. All EV batches induced a significant, dose-dependent reduction in tumor necrosis factor (TNF)-α and interleukin (IL)-6, key pro-inflammatory cytokines [Figure 5A and B]. Notably, at a concentration of 3 × 10^9^ particles/mL, more than 50% inhibition of both TNF-α and IL-6 production was observed, with no statistically significant differences among the three batches. These results indicate that despite minor batch-specific variations in protein abundance, the core, conserved proteomic signature of MSC-EVs, including membrane and transport-related proteins, likely underlies their highly reproducible anti-inflammatory activity and functional consistency in intercellular communication and immune modulation.

Overall, these findings collectively indicate that MSC-EV preparations maintain both molecular and functional stability across independent batches, reinforcing their potential for standardized therapeutic applications. The conserved proteome provides a molecular basis for reproducible potency, supporting the reliability of MSC-EVs in modulating inflammatory responses. Likely, a multitude of proteins and other EV molecules convey the anti-inflammatory effects of MSC-EVs. However, there is a redundancy between batches, by which any combination of molecules in the different MSC-EV batches will convey similar anti-inflammatory properties.

## DISCUSSION

The present study demonstrates that GMP-grade EVs derived from MSCs exhibit remarkable molecular and functional consistency across multiple donors. Using comprehensive proteomic profiling, we identified a robust core proteome comprising more than 80% of total detected proteins that remained unchanged between EV batches, indicating that donor variation exerts only minor differences in the overall protein composition. Guided by our previous finding that MSC-derived EVs retain therapeutic activity even after depletion of cytosolic contents, implying the crucial role of membrane components^[11]^. Importantly, the conserved subset of membrane-associated proteins, comprising adhesion molecules, transporters, and signaling receptors, reflects the stability of key components likely responsible for EV-mediated intercellular communication. This high degree of molecular reproducibility is particularly encouraging for the clinical translation of MSC-EV therapies, where batch-to-batch consistency represents a critical quality parameter for regulatory approval.

Our molecular analyses of EVs partially align with previous studies showing that MSC-EVs retain specific proteomic features regardless of MSC donor origin^[21]^. Comparative analyses of EVs from various MSC sources have demonstrated that nearly half of their proteomes overlap, highlighting a universal MSC-EV signature^[21,22]^. Angulski *et al.* further reported that 60% of proteins were shared between EVs from bone marrow- and umbilical cord-derived MSCs, with many associated with cell maintenance and anti-oxidative stress functions^[22]^. However, no studies have rigorously assessed donor-to-donor variability within the same MSC type under GMP-compliant production conditions, with most focusing primarily on membrane protein profiles. By using standardized culture conditions and TFF on a large scale, our work shows that GMP manufacturing can produce functional EVs with reproducible membrane-associated molecular and biophysical properties, supporting the feasibility of a consistent pipeline for clinical-grade EV therapeutics. Nevertheless, we note some batch-to-batch variability in selected membrane proteins, including CD81 [Figure 1D]. Given that CD81 is a well-established EV-associated tetraspanin involved in vesicle biogenesis and interactions with recipient cells^[23]^, differences in its expression could theoretically influence some EV functions under certain experimental conditions.

In addition to molecular characterization, the functional stability of EVs was confirmed through *in vitro* macrophage assays. All EV batches suppressed OMV-induced TNF-α and IL-6 secretion in a comparable and dose-dependent manner, indicating that the observed proteomic conservation translates into consistent biological efficacy. The reproducibility of anti-inflammatory effects across donors highlights the potential of a conserved proteomic core, particularly membrane proteins involved in integrin signaling and cytokine modulation, to mediate EV therapeutic functions. Previous studies have shown that MSC-EVs can induce prostaglandin E2 (PGE2) production in macrophages, contributing to their polarization toward an anti-inflammatory phenotype^[24,25]^. Accordingly, the anti-inflammatory activity of the GMP-grade MSC-EVs described here may also be mediated, at least in part, through macrophage PGE2 induction.

While our findings highlight the functional relevance of a conserved core proteome, this study primarily identifies conserved membrane proteins and reports associations, rather than pinpointing the specific EV components responsible for anti-inflammatory effects. One notable candidate that has been discussed is CD73, a membrane-bound ecto-5’-nucleotidase. It mediates anti-inflammatory responses largely through adenosine generation and subsequent suppression of immune cell activity^[26,27]^. Therefore, it is possible that CD73, along with other conserved membrane proteins, contributes partially to the observed EV-mediated anti-inflammatory effects. Although establishing a direct mechanistic link between specific membrane proteins and functional outcomes was beyond the scope of this study, CD73 represents a promising target for future mechanistic investigations.

As mentioned above, we focused on a standardized macrophage-based assay to link conserved vesicular proteins to a reproducible anti-inflammatory potency test suitable for donor-to-donor comparison. Notably, the same GMP-grade EV batches have also been evaluated in an independent, disease-relevant model of T-helper type 2 (Th2) inflammation in human keratinocytes mimicking atopic dermatitis^[12]^. In that study, EVs from all donors consistently suppressed IL-4/IL-31 cytokine responses, demonstrating conserved immunomodulatory activity across donors and in a distinct cell type. These findings suggest that the EV preparations characterized here exert broader functional effects beyond macrophage cytokine suppression.

Comparative lipidomic analysis is likewise relevant for understanding EV immunomodulatory mechanisms. Our previous comparison of multiple GMP-grade EV batches identified five major lipid classes with no significant inter-batch differences^[12]^. Notably, inflammation-regulating eicosanoids^[28]^ were absent from all EV preparations. Although further lipid analyses are warranted, these findings suggest that EV-mediated anti-inflammatory effects are unlikely to be driven primarily by membrane lipid composition, reinforcing our focus on conserved membrane proteins as key functional mediators and therapeutic targets for EV-based clinical applications.

Despite these promising results, certain limitations warrant consideration. MSC-EVs produced under GMP-compatible conditions often display size heterogeneity, which may affect cargo and biological activity. Our isolation strategy prioritized yield and functionality rather than strict subtype separation, representing a study limitation. Future refinement of methods, including size fractionation combined with size-exclusion chromatography, may generate more uniform EV populations and enable analysis of size-dependent phenotypes and therapeutic efficacy. Although this study analyzed EVs from only three GMP-grade MSC donors, it provides an initial quantitative comparison of their membrane proteomes. This analysis lays the foundation for future studies with larger donor cohorts and more diverse tissue origins to validate and generalize the findings.

While proteomic consistency serves as a valuable indicator of EV similarity between batches, additional omics analyses may reveal subtle donor-dependent variations not captured by protein-level measurements alone. Future integrated omics approaches should therefore aim to identify complementary analyses that provide a more comprehensive understanding of these differences. For instance, integrating proteomics with transcriptomics can determine whether observed protein variations are reflected at the messenger RNA (mRNA) level. In addition, targeted analyses of post-translational modifications, such as glycosylation of EV membrane proteins, may reveal functional heterogeneity that influences signaling, targeting, and immune interactions but is not apparent from overall protein abundance. Furthermore, from a functional perspective, *in vitro* macrophage assays provide important insight into anti-inflammatory activity but do not fully replicate the complexity of *in vivo* immune environments. Future studies employing preclinical disease models and integrated omics approaches will be essential to confirm the robustness and translational relevance of GMP-manufactured MSC-EVs.

In conclusion, this study establishes that MSC-derived EVs produced under GMP conditions maintain a highly conserved molecular composition and reproducible anti-inflammatory activity across independent donors. The identification of a stable core proteome, particularly within the membrane protein subset, provides a strong foundation for defining quality attributes and developing standardized potency metrics for EV-based therapeutics. By demonstrating both molecular fidelity and functional consistency, our findings advance the standardization of MSC-EV production and contribute to the broader goal of translating EV-based therapies into clinically reliable and scalable treatments.

## DECLARATIONS

### Availability of data and materials

The authors confirm that the data presented in the article will be available from the corresponding author upon reasonable request.

### Copyright

© The Author(s) 2026.

## References

[1] Li P, Ou Q, Shi S, Shao C (2023). Immunomodulatory properties of mesenchymal stem cells/dental stem cells and their therapeutic applications. Cell Mol Immunol.

[2] Gao F, Chiu SM, Motan DA (2016). Mesenchymal stem cells and immunomodulation: current status and future prospects. Cell Death Dis.

[3] Trigo CM, Rodrigues JS, Camões SP, Solá S, Miranda JP (2025). Mesenchymal stem cell secretome for regenerative medicine: where do we stand?. J Adv Res.

[4] Debnath K, Heras KL, Rivera A, Lenzini S, Shin JW (2023). Extracellular vesicle-matrix interactions. Nat Rev Mater.

[5] Doyle LM, Wang MZ (2019). Overview of extracellular vesicles, their origin, composition, purpose, and methods for exosome isolation and analysis. Cells.

[6] Lightner AL, Sengupta V, Qian S (2023). Bone marrow mesenchymal stem cell-derived extracellular vesicle infusion for the treatment of respiratory failure from COVID-19: a randomized, placebo-controlled dosing clinical trial. Chest.

[7] Wiest EF, Zubair AC (2025). Generation of current good manufacturing practices-grade mesenchymal stromal cell-derived extracellular vesicles using automated bioreactors. Biology.

[8] Palamà MEF, Gorgun C, Rovere M (2025). Batch variability and anti-inflammatory effects of iPSC-derived mesenchymal stromal cell extracellular vesicles in osteoarthritis in vitro model. Front Bioeng Biotechnol.

[9] Kou M, Huang L, Yang J (2022). Mesenchymal stem cell-derived extracellular vesicles for immunomodulation and regeneration: a next generation therapeutic tool?. Cell Death Dis.

[10] Ko SY, Naora H (2020). Extracellular vesicle membrane-associated proteins: emerging roles in tumor angiogenesis and anti-angiogenesis therapy resistance. Int J Mol Sci.

[11] Park KS, Bergqvist M, Lässer C, Lötvall J (2022). Targeting Myd88 using peptide-loaded mesenchymal stem cell membrane-derived synthetic vesicles to treat systemic inflammation. J Nanobiotechnology.

[12] Shin KO, Lee JH, Chae S (2025). Small EVs from adipose-derived MSCs modulate epidermal barrier and inflammation via sphingosine-1-phosphate signaling pathway. J Extracell Vesicles.

[13] Lee JH, Ha DH, Go HK (2020). Reproducible large-scale isolation of exosomes from adipose tissue-derived mesenchymal stem/stromal cells and their application in acute kidney injury. Int J Mol Sci.

[14] Wiśniewski JR, Zougman A, Nagaraj N, Mann M (2009). Universal sample preparation method for proteome analysis. Nat Methods.

[15] Kim DK, Lee J, Kim SR (2015). EVpedia: a community web portal for extracellular vesicles research. Bioinformatics.

[16] Li S, Zhang J, Liu X (2024). Proteomic characterization of hUC-MSC extracellular vesicles and evaluation of its therapeutic potential to treat Alzheimer’s disease. Sci Rep.

[17] Tejeda-Mora H, Leon LG, Demmers J (2021). Proteomic analysis of mesenchymal stromal cell-derived extracellular vesicles and reconstructed membrane particles. Int J Mol Sci.

[18] Priya R, Jain V, Akhtar J (2022). Proteomic profiling of cell line-derived extracellular vesicles to identify candidate circulatory markers for detection of gallbladder cancer. Front Oncol.

[19] Zhang B, Tian X, Hao J, Xu G, Zhang W (2020). Mesenchymal stem cell-derived extracellular vesicles in tissue regeneration. Cell Transplant.

[20] Chen S, Lei Q, Zou X, Ma D (2023). The role and mechanisms of gram-negative bacterial outer membrane vesicles in inflammatory diseases. Front Immunol.

[21] (2019). van Balkom BWM, Gremmels H, Giebel B, Lim SK. Proteomic signature of mesenchymal stromal cell-derived small extracellular vesicles. Proteomics.

[22] Angulski AB, Capriglione LG, Batista M (2017). The protein content of extracellular vesicles derived from expanded human umbilical cord blood-derived CD133^+^ and human bone marrow-derived mesenchymal stem cells partially explains why both sources are advantageous for regenerative medicine. Stem Cell Rev Rep.

[23] Teng F, Fussenegger M (2020). Shedding light on extracellular vesicle biogenesis and bioengineering. Adv Sci.

[24] Hyvärinen K, Holopainen M, Skirdenko V (2018). Mesenchymal stromal cells and their extracellular vesicles enhance the anti-inflammatory phenotype of regulatory macrophages by downregulating the production of interleukin (IL)-23 and IL-22. Front Immunol.

[25] Shimamura Y, Furuhashi K, Tanaka A (2022). Mesenchymal stem cells exert renoprotection via extracellular vesicle-mediated modulation of M2 macrophages and spleen-kidney network. Commun Biol.

[26] Murphy PS, Wang J, Bhagwat SP (2017). CD73 regulates anti-inflammatory signaling between apoptotic cells and endotoxin-conditioned tissue macrophages. Cell Death Differ.

[27] Eichin D, Pessia A, Takeda A (2021). CD73 contributes to anti-inflammatory properties of afferent lymphatic endothelial cells in humans and mice. Eur J Immunol.

[28] Boilard E (2018). Extracellular vesicles and their content in bioactive lipid mediators: more than a sack of microRNA. J Lipid Res.

