# Supplementary material for: Quantitative proteomic and functional comparison of extracellular vesicles from multiple adipose tissue mesenchymal stem cell donors
Source: Extracell Vesicles Circ Nucl Acids. 2026 Mar 30;7(1):425–40. doi: 10.20517/evcna.2025.173 (PMC13074282; doi:10.20517/evcna.2025.173)
Supplement: Supplementary file 1 [file evcna-7-1-425-SupplementaryMaterials.pdf]

Supplementary Materials

Quantitative proteomic and functional comparison of extracellular vesicles from multiple adipose tissue mesenchymal stem cell donors

Kyong-Su Park[1,#], Dae Hyun Ha[2,3,#], Jun Ho Lee[2], Negar Ordouzadeh[1,4], Markus Bergqvist[1], Hyun Ju Lee[2], Ella Shin[2], Byong Seung Cho[2], Jan Lötvall[1]

[1]Krefting Research Centre, Institute of Medicine, Sahlgrenska Academy, University of Gothenburg, Gothenburg 40530, Sweden.

[2]ExoCoBio Exosome Institute (EEI), ExoCoBio Inc., Seoul 08594, Republic of Korea.

[3]Department of Life Science and Research Institute of Natural Sciences, College of Natural Sciences, Hanyang University, Seoul 04763, Republic of Korea.

[4]Exocure Sweden AB, Gothenburg 41126, Sweden.

[#]These authors contributed equally to this work.

Correspondence to: Prof. Jan Lötvall, Krefting Research Centre, Institute of Medicine, Sahlgrenska Academy, University of Gothenburg, Gothenburg 40530, Sweden. E-mail: jan.lotvall@gu.se

Supplementary Table 1. Characterization of donors and the isolated GMP-grade MSCs

	Donor			Virus	Sterility test		MSC surface marker (relative expression, %)	MSC-negative surface marker (relative expression, %)	Differential potency
	Sex	Age (y)	BMI	HBV/HCV/HIV	Endotoxin (EU/mL)	Myco plasma	CD29 / CD90 / CD105	CD31 / CD45 / HLA-DR	Adipocyte / osteoblast / chondrocyte
MSC1	F	22	26.1	-	< 0.5	-	> 80	< 3	+
MSC2	F	29	26.7	-	< 0.5	-	> 80	< 3	+
MSC3	F	28	22.2	-	< 0.5	-	> 80	< 3	+

Three-donor cells tested negative for adventitious viruses and passed sterility testing. MSC markers showed high CD29, CD90, and CD105 expression, and low CD31, CD45, and HLA-DR. All donors demonstrated differentiation into adipocytes, osteoblasts, and chondrocytes, confirming suitability. -, negative; +, positive.

Supplementary Table 2. The list of common EV proteins matched with the top 100 EV proteins in EVpedia database

Accession	Gene Name	Accession	Gene Name	Accession	Gene Name
P14618	PKM	P05556	ITGB1	P62820	RAB1A
P07355	ANXA2	P49327	FASN	P10909	CLUS
P63104	YWHAZ	P08195	SLC3A2	P50990	CCT8
P62258	YWHAE	P04083	ANXA	P22314	UBA1
P07195	LDHB	P30041	PRDX6	Q9H4M9	EHD1
P07737	PFN1	P62873	GNB1	P62826	RAN
P08758	ANXA5	O43707	ACTN4	P50995	ANXA11
P62937	PPIA	Q9Y490	TLN1	P60842	EIF4A1
P60709	ACTB	P51148	RAB5C	O75340	PDCD6
Q00610	CLTC	P61224	RAP1B	P08133	ANXA6
P07900	HSP90AA1	P23526	AHCY	P60660	MYL6
P08238	HSP90AB1	P32119	PRDX2	P17987	TCP1
P04264	KRT1	P69905	HBA1	P07996	THBS1
Q8WUM4	PDCD6IP	P04899	GNAI2	P13645	KRT10
O00299	CLIC1	P78371	CCT2	P53396	ACLY
P11021	HSPA5	P04792	HSPB1	P62879	GNB2
P07437	TUBB	P60953	CDC42	O14818	PSMA7
P35579	MYH9	P21333	FLNA	P11233	RALA
P68104	EEF1A1	P14625	HSP90B1		
P55072	VCP	P37802	TAGLN2		
P05023	ATP1A1	P35613	BSG		
P35908	KRT2	P02786	TFRC		
P61981	YWHAG	P12814	ACTN1		
P51149	RAB7A	P14625	HSP90B1		
